# Respiratory symptoms and pulmonary physiologic outcomes among post-9/11 veterans with blast exposure

**DOI:** 10.3389/fpubh.2026.1753539

**Published:** 2026-05-12

**Authors:** Danielle R. Glick, Joanna M. Gaitens, Clayton H. Brown, Melissa A. McDiarmid, Maxwell Reback, Katherine H. Chin, Kerri L. Cavanaugh, William E. Lawson, Peruvemba Sriram, Lisa Beck, John Duch, Samuel M. Aguayo, Paska Permana, Stella E. Hines

**Affiliations:** 1 Department of Veterans Affairs Medical Center, Baltimore, MD, United States; 2 University of Maryland School of Medicine, Baltimore, MD, United States; 3 Tennessee Valley Health Care System Veterans Affairs Medical Center, Nashville, TN, United States; 4 Malcolm Randall Veterans Affairs Medical Center, Gainesville, FL, United States; 5 Oklahoma City Veterans Affairs Medical Center, Oklahoma City, OK, United States; 6 Audie L. Murphy Veterans Affairs Hospital, San Antonio, TX, United States; 7 Phoenix Veterans Affairs Health Care System, Phoenix, AZ, United States

**Keywords:** blast, diffusion capacity, dyspnea, lung volumes, military, oscillometry, spirometry

## Abstract

**Introduction:**

Among post-9/11 veterans reporting blast exposure from the Veterans Affairs (VA) Toxic Embedded Fragment (TEF) registry, respiratory symptoms are common. Blast injury may cause small airway damage, possibly leading to chronic respiratory pathology. We hypothesized that veterans with blast exposure would have worse pulmonary physiologic testing and be more symptomatic.

**Materials and methods:**

Veterans enrolled in the VA TEF registry completed study questionnaires assessing blast exposure, history of traumatic brain injury (TBI), and respiratory symptoms and diagnoses. Veterans underwent traditional full pulmonary function testing (PFT) and impulse oscillometry (IOs). Results were compared between blast-exposed and unexposed groups and adjusted for covariates. The impact of blast severity was further assessed using surrogates, such as TBI.

**Results:**

Among 401 veterans who completed questionnaires, 361 had blast exposure and were more likely to experience dyspnea. Both exposed and unexposed veterans frequently reported respiratory symptoms such as cough, phlegm, and wheeze. Pulmonary physiologic testing was completed in 369 veterans. Veterans with blast exposure had significantly higher FVC, TLC, and DLCO measures, lower FEV1/FVC, but similar IOs results. When TBI was used as a surrogate for more severe blast, veterans reporting TBI had lower TLC, FRC, RV, and RV/TLC ratio.

**Conclusion:**

Post-9/11 veterans with blast exposure reported more dyspnea but did not have lower lung function values compared to those without. Presence of TBI and blast was associated with lower lung function. Further work is needed to elucidate the mechanism of this association and respiratory impacts of blast injury and TBI.

## 1 Introduction

Military service members and veterans who have deployed to the Middle East and Southwest Asia since 2001 commonly report blast exposure (1–4). Acutely, blast injury can result in immediate pulmonary complications, such as respiratory failure, hemorrhage, pulmonary contusion, or pneumothorax. Other organ systems can also be affected, leading to conditions such as traumatic brain injury (TBI), auditory conditions (tinnitus and hearing loss), acute and chronic sinus disease, post-traumatic stress disorder, and suicide (5). The long-term respiratory effects of blast injury are less well understood. In the face of mounting evidence for lung disease among post-9/11 veterans, in 2020 The National Academies recommended that respiratory health effects from blast should be evaluated (6).

Evidence for the association between blast exposure and adverse respiratory health effects exists both in the mechanistic and clinical epidemiologic literature. Mechanisms of injury may include pressure-induced shearing of the small airway epithelium (7), changes to macrophage polarization (8), and changes to autonomic responses (2, 9, 10). These findings support the biologic plausibility for blast-induced lung disease. Epidemiologic literature has observed more variable findings. Some studies have shown associations between blast and respiratory symptoms (11, 12), forced expiratory volume in one second (FEV1) (13) and lung clearance index (LCI) (14) on pulmonary function testing (PFT). Others, however, have shown no differences in PFTs, functional small airways disease on CT scans, or presence of expiratory central airways collapse based on blast exposure (15–17). As a result, consistent patterns have not emerged, potentially limited by variability of testing, sample sizes, study population comparability, and blast exposure assessment method.

Based on these findings, we sought to further explore the effect of blast exposure on veteran respiratory health. Pulling from the Department of Veterans Affairs Toxic Embedded Fragment Registry (TEF)—part of a national surveillance program to identify and track veterans with retained embedded fragments which can be the result of blast and other sources of injury, such as bullets, vehicular injuries or falls,—we compared veterans with a reported history of blast exposure to those with no blast exposure. We hypothesized that Veterans who sustained exposures to blasts would have a greater prevalence of respiratory health abnormalities than those who had not sustained exposures to blasts. Further, we hypothesized that veterans who experienced other effects from blast, such as blast-induced TBI, tympanic membrane rupture, or sinus disease, would have experienced higher severity blast impact, and that this would correlate with greater prevalence of respiratory health abnormalities.

## 2 Materials and methods

### 2.1 Study population and recruitment

As part of a larger study examining general, renal, and respiratory health effects among veterans at risk for having embedded metal fragments, we identified and recruited eligible veterans from April 2018 through March 2021 from the VA's TEF Registry. Approximately 10% of TEF Registrants report injury from a non-blast source, creating an available pool of Veterans who would otherwise have comparable demographic characteristics (11). A veteran was considered eligible if they reported having a retained fragment, documented by X-ray, that occurred as the result of an injury sustained during deployment to Iraq or Afghanistan. Screening for automatic inclusion in the TEF registry is performed within the VA's electronic medical record by providers nationwide as part of routine clinical care of post-9/11 Iraq and Afghanistan veterans (18). Veterans who received care at one of six VA facilities were included in this study: VA Maryland Health Care System, VA Central Tennessee Valley Health Care System, VA North Florida/South Georgia Veterans Health System, Oklahoma City VA Health Care System, South Texas Veterans Health Care System, or the Phoenix VA Health Care System.

Study recruitment was performed through both direct mailing with follow-up phone calls as well as study flyers distributed by primary care providers. Upon study enrollment, most veterans completed an in-person, one-day visit to their local VA. During this visit, they completed: informed consent, two separate questionnaires—a TEF Registry exposure questionnaire collected as part of usual clinical care which captured details about their injury and a study-specific questionnaire—as well as study-specific full, pre-bronchodilator pulmonary function tests (PFTs) and impulse oscillometry (IOs) testing. Due to the COVID-19 pandemic, in-person visits were suspended in March 2020. Following resumption of research activity in August 2020, pulmonary physiologic testing (PFT and IOs) was not performed due to the risk of respiratory virus transmission.

The study was powered to achieve similar effect sizes for pulmonary function test differences from prior studies examining exposure-based differences (19–21). This led to a predetermined total sample size goal of 396 determined based on embedded metal fragments exposure groups. An earlier questionnaire-based portion of this study was powered to determine blast-associated differences in respiratory symptoms. This embedded fragment population represented a convenience sample to evaluate, additionally, for blast impacts on pulmonary function outcomes. The following entities approved this protocol: VA Central Institutional Review Board (IRB) (protocol #17–13), the US Army Medical Research and Development Command Human Research Protection Office, protocol A-19235, VA Research & Development Committees locally at the Baltimore, Gainesville, Nashville, Oklahoma City, San Antonio, and Phoenix VA Medical Centers and their affiliated IRBs (University of Maryland, Baltimore protocol #HP-00074623).

### 2.2 Survey instrument and exposure definition

Health outcomes, both general and respiratory specific, were assessed using a survey created specifically for use in the present study based on existing questionnaires described in detail elsewhere (22). Demographic and military service information was gathered using questions from the National Health Study for a New Generation of U.S. Veterans (23). Respiratory health was evaluated utilizing the American Thoracic Society (ATS) and Division of Lung Disease (ATS-DLD-78) questionnaire (24). Outcomes of interest from these questionnaires included the presence of cough, wheeze, and dyspnea as well as respiratory diagnoses (such as chronic bronchitis, emphysema, asthma, or other chest illness) and history of smoking and/or vaping.

Exposure to blast was assessed using portions of tools crafted to identify traumatic brain injury as a surrogate marker. To formally assess the presence of blast, we utilized the Brief Traumatic Brain Injury Screening (BTBIS) tool, a scale initially developed to identify service members requiring clinical evaluation for TBI (25). Blast exposure was defined as an affirmative response to the first question of the BTBIS, consistent with our prior work (11). This question asks “did you have any injury(ies) during your deployment from any of the following?”, with a subsequent selection of “blast” among six response options (“a) fragment, b) bullet, c) vehicular (any type of vehicle, including airplane), d) fall, e) blast (improvised explosive device, rocket propelled grenade, land mine, grenade, etc.), f) other (specify)”). The specific presence of TBI was also assessed through self-report (“Have you ever been told you had a traumatic brain injury (TBI)?”). Given that barotrauma from blast exposure can also result in primary injury of air-fluid or air-tissue interfaces such as the tympanic membrane, sinuses, and the chest cavity, veterans were asked whether they had experienced those types of injuries as a result of a blast or explosion (3, 26).

### 2.3 Pulmonary function evaluation

Pre-bronchodilator IOs, spirometry, lung volume, and diffusion capacity testing were performed for all participants, in that order. Testing was performed at each site by Respiratory Therapists trained according to ATS and European Respiratory Society (ERS) guidelines (23–26). Study staff at a central site monitored physiology testing results for quality, as we have previously described (22, 27–30). The following results were collected, by test: spirometry—forced expiratory volume in 1 second (FEV_1_), forced vital capacity (FVC), FEV_1_/FVC ratio, forced expiratory flow at 25%−75% (FEF_25%−75%_); lung volumes – total lung capacity (TLC), functional residual capacity (FRC), residual volume (RV), RV/TLC ratio; diffusing capacity of carbon monoxide (DLCO); IOs—resistance at 5 Hz (R_5_), resistance at 20 Hz (R_20_), frequency dependence of resistance (R_5 − 20_), reactance at 5 Hz (X_5_), resonant frequency (Fres), area of reactance (AX), coherence at 5 Hz, coherence at 10 Hz, and coefficients of variation (CV) for R_5_, R_20_, and Fres. IOs testing at all sites was performed on the Vyaire IOs system, while PFTs were performed at each site's clinical PFT labs using either Vyaire (3 sites), Morgan Scientific, MGC Platinum Elite, and Inspire HD (1 site each) systems. At all sites, IOs testing was performed prior to PFTs.

All participants underwent pulmonary function evaluation with the exception of 33 individuals who were recruited after the onset of the COVID-19 pandemic in March 2020. After a period of full cessation of research activities at that time, recruitment resumed in August 2020 with a modified protocol that excluded the performance of PFTs and IOs.

### 2.4 Data analysis

Analyses were performed using SAS version 9.4 and R version 4.5.3.

#### 2.4.1 Demographics

Demographic, military, and occupational characteristics overall and stratified by blast exposure were described with means and standard deviations for continuous variables and counts and percentages for categorical variables. Chi-square and *t*-tests were used, as appropriate, to identify significant differences between the two exposure groups.

#### 2.4.2 Comparison of symptoms and diagnoses

In assessing respiratory health outcomes, the presence of respiratory symptoms was determined by the self-reported presence of any usual cough, wheeze, or dyspnea, assessed as defined in our previous work (22). Among diagnoses, presence of COPD was determined by self-report of ever having a doctor-confirmed diagnosis of chronic bronchitis or emphysema. Presence of asthma was queried in different ways, in increasing order of presumed specificity and severity: (1) ever had and still having asthma; (2) having asthma confirmed by a doctor; and (3) having asthma and currently requiring medicine or treatment. For a summary variable inclusive of any confirmed COPD or asthma, participants who met any of the COPD or asthma criteria were combined for analysis. Participants could also select “other chest illness” as a diagnosis, with an option to include a free text explanation. To compare prevalence of symptoms and diagnoses between the blast exposed and unexposed groups, logistic regression was used. Adjustment was made for the covariates of age, race, sex, height, weight, smoking, vaping, dust exposure (defined as working 1 year or more in a moderate or severely dusty job), and time since reported injury.

#### 2.4.3 Comparison of pulmonary physiologic testing

Mean values of both PFT and IOs outcomes between the blast exposed and unexposed groups were compared using multiple linear regression adjusting for the same covariates described above. Percent of predicted values for PFT results were calculated in reference to recommendations of the ATS and ERS (31–33). Patterns of abnormality on pulmonary physiology testing were also assessed. In PFT testing, patterns were identified on spirometry, lung volumes, and diffusion by comparing individual values against the reference values of upper and lower limits of normal (ULN and LLN) defined by the Global Lung Initiative (32–34). For IOs values, these were compared to the reference values of Vogel and Smidt, as predetermined at the time of study initiation as being the largest, most technically similar to our study population (35). As previously described, we set cut points of abnormality as: total airways resistance (R_5_) >150% predicted, R_5 − 20%_ >20%−30% and ≥30%, and X_5_ < −0.1176 kPa/L/s (22).

#### 2.4.4 Effect of blast impact severity on pulmonary physiology outcomes

To assess the effect of blast severity on pulmonary physiologic outcomes, we analyzed outcomes only among those who reported blast exposure but stratified by different markers of blast impact. Multiple linear regression was used to compare PFT and IOs outcome mean measured values between self-reported levels of blast-induced loss of consciousness (LOC), and between those who had and had not been diagnosed with TBI, blast-induced tympanic membrane rupture, and blast-induced sinonasal problems. This analysis was adjusted for the following covariates: age, race, sex, height, weight, pack-years of smoking, vaping, years since injury, and distance from blast.

## 3 Results

A total of 402 veterans participated in the study, of which 361 were considered blast exposed and 41 blast unexposed, of which 401 also provided information about respiratory health outcomes. Of these, 369 completed pulmonary physiology testing, including both PFT and IOs. Table 1 shows the demographic and military characteristics of the group as a whole and classified by blast exposure. The blast exposure groups were similar regarding body composition, sex (mostly male), race (mostly white), and service branch (mostly Army). They also shared a similar length of time since injury (mean (SD) 12.8 (2.8) years). Smoking by distribution of pack-years was similar between the groups, as well as prevalence of vaping. Most participants had never vaped and smoked for 10-pack years or less. The groups differed in the prevalence of having worked in moderately to severely dusty jobs, with those reporting blast more likely to have experienced this.

**Table 1:** Demographic and military characteristics of Veterans with and without blast exposure.

Characteristic	Full cohort	No blast exposure	Blast exposed	*P*-value
	(*n* = 402)	(*n* = 41)	(*n* =361)	
	Mean (SD) or *n* (%)	Mean (SD) or *n* (%)	Mean (SD) or *n* (%)	
**Age**	41.7 (7.7)	41.9 (9.4)	41.7 (7.5)	0.89
**Height (cm)**	176.7 (7.5)	176.4 (7.1)	176.7 (7.5)	0.83
**Weight (kg)**	96.9 (19.3)	93.7 (18.8)	97.3 (19.4)	0.29
**Body mass index (BMI)**	31.0 (5.7)	30.1 (6.1)	31.1 (5.6)	0.39
**Sex**				0.51
Male	384 (95.5)	40 (97.6)	344 (95.3)	
Female	18 (4.5)	1 (2.4)	17 (4.7)	
**Race/ethnicity**				0.81
White	254 (63.2)	26 (63.4)	228 (63.2)	
Hispanic	73 (18.2)	9 (22.0)	64 (17.7)	
Black	32 (8.0)	2 (4.9)	30 (8.3)	
Other	43 (10.7)	4 (9.8)	39 (10.8)	
**Service branch**				0.41
Army	299 (74.4)	29 (70.7)	270 (74.8)	
Marines	71 (17.7)	10 (24.4)	61 (16.9)	
Other	32 (8.0)	2 (4.9)	30 (8.3)	
**Years since injury**				0.09
10 years or less	56	9 (23.1)	47 (13.2)	
>10 years	339	30 (76.9)	309 (86.8)	
**Smoking (pack-years)**				0.76
0	176 (45.6)	18 (46.2)	158 (45.5)	
>0–10	116 (30.1)	10 (25.6)	106 (30.6)	
>10	94 (24.4)	11 (28.2)	83 (23.9)	
**Ever vaped**	57 (14.3)	6 (14.6)	51 (14.2)	0.94
**Work for a year or more in a dusty job**				**0.048**
< 1 year or mild	235 (58.8)	30 (73.2)	205 (57.1)	
Moderate or Severe	165 (41.3)	11 (26.8)	154 (42.9)	

Bold values indicate clinical significance.

Prevalences of respiratory symptoms and diagnoses are presented in Table 2. Veterans with blast exposure did not differ from those without it in respect to the presence of cough, phlegm, and wheeze. However, those with blast exposure were significantly more likely to have shortness of breath when hurrying on level ground or walking up a slight hill, or when walking with others of their same age. Overall, both the blast exposed and unexposed groups frequently reported having “any symptoms” (79.1% overall prevalence). Neither prevalence of “any symptoms” nor diagnoses differed between the blast exposure groups.

**Table 2:** Respiratory symptoms and diagnoses in Veterans in the Toxic Embedded Fragment Registry with and without reported blast exposure.

Respiratory outcome	Total	No blast	Blast	adjusted *p* value^*^	aOR^*^
	*N* = 401	*N* = 41	*N* = 353		
	*N* (%)	*N* (%)	*N* (%)		
SYMPTOMS					
Cough (4–6x/day, 4+ days/week)	183 (46.4)	19 (46.3)	164 (46.5)	0.978	1.01 (0.46–2.23)
Phlegm (2xd, 4+day/week)	172 (44.0)	17 (41.5)	156 (44.6)	0.971	0.99 (0.45–2.18)
Wheezing					
Persistent wheeze	226 (56.4)	20 (48.8)	206 (57.2)	0.970	1.02 (0.47–2.21)
Severe wheeze	112 (29.2)	9 (22.5)	103 (29.9)	0.230	1.86 (0.68–5.11)
Shortness of breath (SOB)				
SOB when hurrying on the level or walking up slight hill	277 (69.3)	23 (56.1)	255 (70.8)	**0.040**	**2.35 (1.04–5.33)**
Walk slower than people of same age due to SOB	155 (38.7)	9 (22.0)	146 (40.6)	**0.010**	**4.26 (1.41–12.83)**
Stop for breath walking at own pace on level	128 (32.0)	11 (26.8)	117 (32.5)	0.250	1.75 (0.68–4.52)
Too breathless to leave house or with dressing/undressing	59 (14.7)	6 (14.6)	53 (14.7)	0.244	2.48 (0.54–11.42)
Any symptoms	315 (79.1)	32 (78.1)	283 (79.3)	0.986	0.99 (0.39–2.55)
DIAGNOSES					
COPD confirmed by a doctor	31 (8.1)	1 (2.5)	30 (8.7)	0.320	2.86 (0.36–22.70)
Asthma (still have)	26 (6.8)	3 (7.5)	23 (6.7)	0.311	0.50 (0.13–1.92)
Asthma confirmed by a doctor	51 (13.2)	5 (12.5)	46 (13.3)	0.494	0.69 (0.24–1.99)
Asthma—currently require medicine or treatment	21 (5.7)	3 (7.7)	18 (5.4)	0.149	0.36 (0.09–1.45)
Any confirmed COPD or current confirmed Asthma	46 (12.4)	4 (10.3)	42 (12.7)	0.619	0.75 (0.24–2.37)
Other chest illness	32 (8.0)	3 (7.3)	29 (8.1)	0.409	0.57(0.15–2.19)

^*^Adjusted for sex, age, race, height, weight, pack-years of smoking, dusty job, vaping, and years since injury. Persistent wheeze = Any YES to any of the following: wheeze occasionally apart from colds, most days and nights. Severe wheeze = Any YES to any of the following: ever have attacks of wheezing making you feel shortness of breath, had 2 or more such episodes of wheezing causing shortness of breath, required meds for attacks of wheezing. Bold values indicate clinical significance.

Table 3 shows the mean measured and percent of predicted values on PFT and IOs testing in the group overall and between the blast exposed and unexposed groups. Mean measured values were statistically significantly higher in the blast exposed group for FVC, TLC, and DLCO, while FEV1/FVC ratio was lower in the blast group. On IOs testing, R_5_ in each group was borderline elevated (>150% predicted), as was R_20._ AX and Fres values were above the thresholds for abnormal (>0.33 kPa/L/sec and >12 Hz, respectively). Between exposure groups, however, there was no significant difference in these testing outcomes.

**Table 3:** Pulmonary function test (PFT) and impulse oscillometry (IOs) values in Toxic Embedded Fragment Registry Veterans with and without reported blast exposure.

	Total with study	No blast	Blast	adjusted *p* value^†^
Traditional PFTs	Mean ±SD	Mean ±SD	Mean ±SD	
Spirometry^*^	*N* = 369	*N* = 35	*n* = 334	
**FEV1, L -**	3.75 ± 0.77	3.65 ± 0.55	3.76 ± 0.79	0.202
**FEV1, % predicted**	96.9 ± 15.6	95.2 ±10.6	97.1 ± 16.1	0.428
**FVC, L**	4.79 ± 0.95	4.62 ± 0.72	4.81 ± 0.97	**0.010**
**FVC, % predicted**	100.4 ± 15.0	98.0 ± 13.4	100.6 ± 15.2	0.095
**FEV1/FVC, %**	78.39 ± 5.93	79.43 ± 6.30	78.28 ± 5.89	**0.010**
**FEF25–75, L/s**	3.54 ± 1.08	3.51 ± 0.97	3.54 ± 1.10	0.570
**FEF25–75, % predicted**	91.9 ± 26.0	92.4 ± 26.1	91.8 ± 26.1	0.641
Lung volumes^**^	*n* = 366	*n* = 35	*n* = 331	
**TLC, L**	6.43 ± 1.15	6.12 ± 0.86	6.46 ± 1.17	**0.024**
**TLC, % predicted**	90.9 ± 13.1	87.1 ± 9.9	91.3 ± 13.3	**0.040**
**FRC, L**	2.82 ± 0.83	2.80 ± 0.81	2.82 ± 0.83	0.433
**FRC, % predicted**	84.9 ± 23.1	84.5 ± 22.7	84.9 ± 23.1	0.467
**RV, L**	1.57 ± 0.60	1.46 ± 0.53	1.58 ± 0.61	0.709
**RV, % predicted**	94.4 ± 33.9	85.7 ± 25.7	95.3 ± 34.5	0.210
**RV/TLC, %**	24.32 ± 7.60	23.60 ± 6.74	24.40 ± 7.69	0.480
**RV/TLC, % predicted**	103.8 ± 30.1	99.1 ± 25.0	104.3 ± 30.5	0.688
Gas exchange	*n* = 367	*n* = 35	*n* = 332	
**DLCO** ^***^ **ml/min/mmHg**	28.76 ± 6.12	27.65 ± 6.48	28.87 ± 6.08	**0.041**
**DLCO, % predicted**	94.8 ± 17.4	92.1 ± 22.4	95.1 ± 16.8	0.372
**Impulse oscillometry**				
Resistance	*N* = 369	*N* = 35	*N* = 334	
**R5, kPa/L/sec**	0.412 ± 0.128	0.418 ± 0.138	0.411 ± 0.127	0.467
**R5, % predicted**	150.4 ± 47.36	154.8 ± 52.67	149.9 ± 46.83	0.409
**R20, kPa/L/sec**	0.337 ± 0.092	0.351 ± 0.104	0.336 ± 0.091	0.148
**R20, % predicted**	141.99 ± 38.18	148.9 ± 46.39	141.3 ± 37.22	0.108
**R5–20, %**	16.67 ± 9.83	14.85 ± 10.07	16.86 ± 9.8	0.178
Reactance	*N* = 369	*N* = 35	*N* = 334	
**X5, kPa/L/sec**	−0.121 ± 0.059	−0.122 ± 0.064	−0.121 ± 0.059	0.445
**AX, kPa/L/sec**	0.613 ± 0.641	0.595 ± 0.766	0.615 ± 0.629	0.929
**Fres, Hz**	14.62 ± 4.73	13.79 ± 4.89	14.71 ± 14.71	0.288

^†^Adjusted for sex, age, race, height, weight, pack-years of smoking, ever vaping, dusty job, and time since injury. ^*^Spirometry adjusted comparisons include 353 participants; ^**^Lung Volume adjusted comparisons includes 350 participants ^***^DLCO adjusted comparisons includes 351 participants. Bold values indicate clinical significance.

Table 4 further explores PFT and IOs outcomes by patterns of abnormalities. Most veterans had normal spirometry (84% overall), with no significant differences between exposure groups in presence of obstructive or restrictive spirometry patterns. Lung volume patterns were also typically normal (75.1% overall), with evidence of hyperinflation only in the blast exposed group (5.4%). Diffusing capacity was also normal overall (83.9%), however those exposed to blast were significantly more likely to be normal (85.8% exposed vs. 65.7% unexposed), while unexposed veterans showed increased (8.6%) or reduced (25.7%) values more often.

**Table 4:** Prevalence and odds of pulmonary function test (PFT) and impulse oscillometry (IOs) abnormalities compared to normal patter in Toxic Embedded Fragment Registry Veterans with and without reported blast exposure.

Pattern	Total	No blast	Blast	*p*-value	Odds ratio (95% CI)
		(*n* = 35)	(*n* = 334)		
PFT abnormality	*N* (%)	*N* (%)	*N* (%)		
Spirometry pattern (*n* = 369)				0.360 (overall difference)	
**Normal (FEV**_**1**_**/FVC** **≥LLN and FVC** **≥LLN)**	310 (84.0)	27 (77.1)	283 (84.7)	–	–
**Obstructive (FEV**_**1**_**/FVC** **<** **LLN)**	29 (7.9)	3 (8.6)	26 (7.8)	0.767	0.83 (0.24–2.91)
15.6-7.5,-14.1499pt**Restrictive (FEV**_**1**_**/FVC** **≥LLN and FVC** **<** **LLN)**	30 (8.1)	5 (14.3)	25 (7.5)	0.162	0.48 (0.17–1.35)
Lung volume pattern (*n* = 365)				0.181 (overall difference)	
**Normal (LLN** **≤TLC** **≤ULN and RV** **≤ULN)**	274 (75.1)	25 (71.4)	250 (75.5)	–	–
**Hyperinflated or air trapping (TLC** **>** **ULN or** **LLN** **≤TLC** **≤ULN and RV** **>** **ULN)**	18 (4.9)	0	18 (5.4)	0.987	∞
**Restrictive (TLC** **<** **LLN)**	73 (20.0)	10 (28.6)	63 (19.0)	0.248	0.63 (0.29–1.38)
Gas exchange pattern (*n* = 367)				0.007 (overall difference)	
**Normal (LLN** **≤DLCO** **≤ULN)**	308 (83.9)	23 (65.7)	285 (85.8)	–	–
**Reduced (DLCO** ** < LLN)**	47 (12.8)	9 (25.7)	38 (11.5)	**0.012**	0.34 (0.15–0.79)
**Elevated (DLCO** **>** **LLN)**	12 (3.3)	3 (8.6)	9 (2.7)	**0.043**	0.24 (0.06–0.96)
IOs abnormality				adjusted *p*-value^*^	Adjusted odds ratio^*^(95% CI)
Proportion with abnormality (*n* = 369)					
**Abnormal R5 (R5>150% pred)**	163(44.3)	17 (48.57)	146 (43.84)	0.2356	0.62 (0.28–1.37)
**Abnormal R5–20 (≥20 % but** ** < 30%)**	81 (22.0)	6 (17.1)	75 (22.5)	0.1159	2.50 (0.80–7.81)
**Abnormal R5–20 (≥30%)**	38 (10.3)	2 (5.71)	36 (10.8)	0.2586	2.46 (0.52–17.71)
**Abnormal X5 (X5 < )**–**0.1176 kPa/L/s**	168 (45.5)	17 (48.6)	151 (45.2)	0.3274	0.67 (0.29–1.50)

^*^Adjusted for sex, age, race, height, weight, pack-years of smoking, ever vaping, dusty job, and time since injury. Bold values indicate clinical significance.

While overall patterns of IOs were normal, the prevalence of abnormality on this test was higher than on PFTs. R_5_ was above the normal threshold in 44.3% overall, with similar but not significantly different prevalence in the exposure groups. Frequency dependence of resistance (R_5 − 20_ ≥30%), an indicator of obstruction, was present in only 10.3% overall with similar findings in the exposure groups. Finally, reactance at 5 Hz (X_5_) showed similar prevalence of abnormality as R_5_, with 45.5% of the overall group being abnormal.

We also examined lung function testing outcomes between marker levels of severity of blast impact (Table 5). This was quantified as self-reported blast-induced LOC (4 levels), tympanic membrane rupture, or sinonasal problems, as well as the presence of a self-reported TBI diagnosis. Veterans reporting a diagnosis of TBI demonstrated lower TLC (*p* = 0.04), FRC (*p* = 0.003), RV (*p* = 0.003), and RV/TLC ratio (*p* = 0.014) in adjusted analysis. Similarly, when compared to those without the stated injury, those with reported blast-induced tympanic membrane rupture had lower RV/TLC (*p* = 0.014), and those with blast-induced sinonasal problems had lower FRC (*p* = 0.01). When examining the effect of blast on IOs outcomes, only R_5_ showed any significant effect, with mild LOC associated with higher resistance and, interestingly, the presence of blast-induced sinonasal problems resulting in a lower resistance.

**Table 5:** Regression coefficients and significance values in blast-exposed Veterans, stratified by markers of blast severity and adjusted for covariates.

PFT/IOS value	Blast-induced loss of consciousness (LOC)				Presence of self-reported TBI diagnosis		Blast-induced tympanic membrane rupture		Blast-induced sinonasal problems	
	None	Mild	Moderate	Severe	Absent	Present	Absent	Present	Absent	Present
Responses *N* (%)	17 (5.1)	189 (56.6)	94 (28.1)	34 (10.2)	91 (27.3)	242 (72.7)	245 (73.4)	89 (26.7)	130 (38.9)	204 (61.1)
PFT value: estimate (p^*^)										
**FEV**_**1**_	0	−0.285 (0.09)	−0.317 (0.06)	−0.329 (0.08)	0	−0.004 (0.96)	0	0.137 (0.08)	0	−0.030 (0.67)
**FVC**	0	−0.342 (0.07)	−0.355 (0.07)	−0.39 (0.07)	0	−0.025 (0.78)	0	0.112 (0.20)	0	−0.072 (0.37)
**FEV**_**1**_ **/FVC**	0	−0.008 (0.62)	−0.009 (0.56)	−0.008 (0.63)	0	0.003 (0.67)	0	0.012 (0.08)	0	0.005 (0.47)
**FEF**_**25 − 75%**_	0	−0.486 (0.087)	−0.486 (0.09)	−0.462 (0.15)	0	0.037 (0.79)	0	0.229 (0.083)	0	−0.054 (0.66)
**TLC**	0	−0.225 (0.38)	−0.388 (0.14)	−0.397 (0.17)	0	**−0.247 (0.04)**	0	−0.039 (0.73)	0	−0.126 (0.24)
**FRC**	0	0.016 (0.94)	−0.122 (0.56)	−0.055 (0.811)	0	**−0.281 (0.003)**	0	−0.087 (0.35)	0	**−0.219 (0.01)**
**RV**	0	0.062 (0.71)	−0.035 (0.84)	−0.043 (0.82)	0	**−0.227 (0.003)**	0	−0.148 (0.05)	0	−0.013 (0.32)
**RV/TLC**	0	1.31 (0.53)	0.914 (0.67)	0.412 (0.86)	0	**−2.38 (0.014)**	0	**−2.06 (0.03)**	0	−0.225 (0.78)
**DLCO**	0	−1.44 (0.31)	−1.71 (0.24)	−2.01 (0.21)	0	−1.00 (0.14)	0	0.833 (0.21)	0	−1.07 (0.07)
IOs value: estimate (p^*^)										
**R**_**5**_	0	0.052 (0.10)	0.033 (0.30)	0.026 (0.46)	0	−0.001 (0.93)	0	−0.01 (0.47)	0	−0.012 (0.38)
**R**_**5%−20%**_	0	1.67 (0.50)	−0.085 (0.97)	0.267 (0.92)	0	−0.192 (0.87)	0	0.270 (0.81)	0	0.514 (0.62)
**X**_**5**_	0	−0.011 (0.46)	0.002 (0.88)	−0.002 (0.90)	0	0.003 (0.70)	0	0.003 (0.64)	0	0.009 (0.16)
**AX**	0	0.203 (0.21)	0.034 (0.84)	0.055 (0.77)	0	−0.019 (0.81)	0	−0.010 (0.90)	0	−0.054 (0.45)

^*^Adjusted for age, race, sex, height, weight, pack—years of smoking, vaping, years since injury, distance from blast. ^†^mild LOC = being dazed or confused, not remembering the injury, or LOC for < 1 min; moderate defined as LOC for 1–20 min; and severe as LOC over 20 min. Bold values indicate clinical significance.

## 4 Discussion

To assess whether blast exposure is associated with adverse respiratory health effects, we compared veterans with and without blast exposure based on a) respiratory symptoms and diagnoses obtained from a questionnaire and b) pulmonary physiology test results from traditional function and impulse oscillometry testing. When assessed using affirmative responses to the BTBIS to signify blast-exposure, we found few differences in blast exposed compared to unexposed veterans regarding respiratory symptoms, except for increased mild dyspnea among those with blast exposure. However, this cohort of young veterans overall expressed a high prevalence of respiratory symptoms without a similarly high prevalence of respiratory diagnoses. Blast exposed veterans had higher FVCs, TLCs, and DLCO values compared to veterans without blast exposure, reflecting better lung function. We also did not observe greater elevated airways resistance on IOs in the blast exposed population compared to the unexposed, or significantly higher evidence for hyperinflation or air-trapping on lung volumes. Thus, by classifying blast exposure according to responses from the BTBIS, we observed few associations with adverse clinical respiratory health outcomes.

We did, however, see statistically significant associations between diagnosis of TBI among blast-exposed veterans and lower values on all lung volumes. TBI was analyzed as a surrogate measure for blast impact severity, and association of TBI with respiratory health outcomes in absence of blast was not the primary aim of this analysis. With other surrogate measures for severity of blast impact, we also saw statistical associations between lower lung volume measurements and sinonasal problem and ear drum rupture. Our findings of associations between TBI and reported abnormal respiratory health are consistent with a growing body of literature showing similar patterns (11, 12, 36–38). With this increasing finding, new questions arise regarding the impact of blast itself on respiratory health or whether downstream effects after neurologic injury might manifest in the lungs.

Reasons that the blast exposed veterans in this population displayed better lung function than the unexposed veterans are unclear. It may be that those who sustained a blast ultimately received more immediate treatment in a medical facility and attention to care, compared to those who did not. In a previous study, we assessed whether treatment at Landstuhl, a military base used for treatment for more severe injuries, impacted experience of respiratory symptoms. We found that respiratory symptoms and diagnoses were less frequently reported among those treated at Landstuhl. Thus, experience of medical care may mitigate some of the long-term health consequences from blast exposure (11). Alternately, our exposure assessment tool, the BTBIS, which is meant to identify individuals at risk for TBI—not respiratory disease, may be sensitive enough to identify exposure groups for self-reported outcomes, but insensitive in classifying groups to identify adverse respiratory health impacts. With respect to lung function, a prior study among treatment-seeking Veterans where blast was assessed based on medical record abstraction found no associations between any PFT or oscillometry parameters and blast exposure as documented in clinical notes. However, average measured and percent of predicted values were higher, although not statistically significantly so, in that study for FVC, TLC, and DLCO among veterans with single mild blast compared to no blast exposure (4.98 L vs. 4.69 L, 6.60L vs. 6.21 L, and 26.67 vs. 25.13, respectively (16). However, this study population also included TBI as one marker of blast exposure. Although directionally similar to our study's PFT findings, impact of blast exposure on lung function in absence of TBI remains obscure.

Recent observational research highlights the potential complexity of the role blast and overpressure exposure may play in a respiratory health outcome. Among service members and veterans evaluated in the Millennium Cohort Study, Belding et al. demonstrated differences in organ system outcomes based on type of blast exposure, where those with high-level blast (“overpressure generated by incoming munitions”) reported more asthma and chronic bronchitis while those with low-level blast (“overpressure generated by outgoing munitions”) experienced more sinus disease (5). Similarly, a study by Englert et al. demonstrated differences in respiratory symptoms among US Marines experiencing TBI after blast- vs. impact-related injuries (39). Those with impact-related TBI (i.e., where physical impact resulted in injury) were more likely to report chest pain, trouble breathing, and persistent cough while those with blast-related TBI more commonly reported tinnitus and difficulty hearing. These relationships for chest and auditory symptoms with blast vs. impact-related TBI were not differentially associated with presence or absence of PTSD. These findings suggest that there may be differences in neurologic sequela from brain injury induced by blast vs. that from a direct impact to the head, with downstream impacts on the respiratory system (39). In an animal model, TM rupture and substantial neuropathological changes in the brain were observed following blast-induced pressure through the ear canal (40). These findings may differ from those occurring following a direct impact to the brain, despite both injuries leading to TBI. Given the increasing consistency in associations between TBI and abnormal respiratory symptoms, coupled with the variable associations between “blast exposure” and adverse respiratory outcomes, there may be key exposure features not yet clearly defined and therefore not discretely assessed that nonetheless play critical roles in the development of abnormal respiratory health.

Our study has several limitations. First, we were limited in our analysis due to our small sample of participants without blast exposure, which was an inherent condition of the study based on recruitment exclusively from the TEF registry (11). The TEF registry includes over 27,000 participants, including those with bullet-only injuries who did not experience a blast (11, 41). Thus, this registry does include a blast unexposed population available as a reference. Additionally, although we obtained our targeted enrollment of participants, 8% of our total sample size did not undergo PFTs/IOs due to research restrictions imposed during the COVID-19 pandemic onset that coincided with our final months of enrollment. Therefore, we may have observed different outcomes had we been able to recruit a larger population of blast unexposed participants. Despite this small unexposed sample size, we had 80% power to detect a medium effect size (Cohen's *d* = 0.51) in our comparison of mean values with our sample sizes of 35 and 334 (Table 3). For our comparison of proportions, there was 80% power to detect a “medium” effect size difference of proportions, for example 0.35 vs. 0.15 (Cohen's *h* = 0.47). Thus, while we may have been unable to detect small differences between groups, such differences might not be clinically significant.

Second, our study relies on self-reported data without verification through medical records or imaging. Participants were recruited from the TEF registry, consisting of veterans who sought VA care, which may not represent the broader post-9/11 veteran population. While our findings may not be generalizable to all blast-exposed individuals, they likely reflect the impact on those with severe blast-related consequences, such as confirmed TBI diagnoses, who are under medical care. Recall bias may affect these self-reported responses. Respiratory symptoms were commonly reported in our participants (79% any, 69% dyspnea). This is similar to observations from a prior questionnaire-based study among TEF Registrants, where respiratory symptoms were reported by 68% of participants (11). While high, our participants' responses were also similar to those of AHOBPR participants, who reported a 61% prevalence of dyspnea or exercise intolerance, with 65% of blast-exposed and 47% of non-blast-exposed registrants reporting these symptoms (12). Among AHOBPR participants who also had PFTs performed for clinical purposes, 68% reported dyspnea, yet 49% had normal spirometry (42). In our study, 69% reported similar levels of dyspnea, yet 84% had normal spirometry, performed for research purposes. Thus, we observe comparable symptom patterns in another veteran registry population, yet more discrepant pulmonary function test results. Discrepancies between burdens of respiratory symptom reporting and pulmonary function abnormalities have been consistently noted in post-9/11 veterans, for unclear reasons. Complexities of perception of dyspnea likely contribute, affected by numerous factors both within and outside of the pulmonary system, including cardiovascular and mental health impacts (43, 44).

Third, we assessed blast using the BTBIS self-reported affirmative responses about blast-induced injury or a positive screen for TBI, regardless of whether the veteran self-reported a diagnosis of TBI. We did this to be most inclusive of potentially blast-exposed individuals, although this exposure assessment may not be specific enough to discriminate effects specific to blast. Additionally, part of the BTBIS differentiates level of consciousness in remembering or not remembering the injury, which may limit ability to rely on self-report of a blast exposure (16, 45). Use of the BTBIS may have created exposure misclassification, and our binary classification of blast exposed vs. unexposed may obscure dose-response relationships that we were unable to demonstrate beyond our severity analysis surrogates. We are unable to determine whether this may have resulted in bias toward the null. Currently, no consensus exists regarding the best tools to assess self-reported blast exposure, although tools are being developed (11, 46). Given the lack of best practice, the use of the BTBIS, a validated TBI screening tool, was deemed appropriate (25).

The use of the BTBIS scale also limits the ability to classify the type and severity of traumatic brain injury suffered. First, regarding type of injury, we included only those individuals who reported blast on the BTBIS, though it is possible they experienced other injuries resulting in TBI as well (for example, blunt trauma). Furthermore, blast injury is a heterogenous exposure with a variety of types and severity, including primary (direct effects from over- and under-pressurization, such as pulmonary barotrauma and concussion), secondary (penetrating trauma), tertiary (including both open and closed brain injuries), and quaternary/quinary (injuries resulting in subsequent exposures such as toxic inhalational injury) 0.2, (5, 47, 48). Although we used surrogates of injury severity, we did not use a standardized injury severity score (as is often used in trauma research) to stratify groups and, by nature of self-report, we are unable to stratify the type of blast experienced (49). However, we did incorporate other markers reflecting severity of injury and type of blast exposure, such as TBI, TM rupture and blast-induced sinus disease (26). While this measure did show associations with PFTs, it may be overly broad, aggregating “blast” to a single exposure and potentially obscuring a graded relationship. For example, Praveen et al. saw differences in lung function among those with high injury severity scores as compared to uninjured (13). If this is the case, we may have overclassified individuals with less severe blast exposure and therefore lacked the sensitivity to detect changes in lung function.

Finally, this cross-sectional study can only show associations between health outcomes and measures of blast exposure. This study is unable to address causation, which requires different study methods and accumulation of a total body of scientific evidence. Thus, we are unable to draw any conclusions about initiation or progression of health effects from blast exposure.

Our study has unique strengths. First, given the uncertainty in best strategies to assess blast retrospectively, we incorporated measures that other researchers have found useful in documenting acute and chronic clinical consequences of blast exposure, such as blast-induced TM rupture, sinus disease, LOC, and memory loss (5, 36, 40). Additionally, we incorporated distance from blast as a covariate in analysis of PFT and IOs results, which has been important in other studies as a predictor of blast impact (10). Finally, we adjusted for smoking exposures and analyzed a full suite of pulmonary physiology measures, including lung volumes, diffusion capacity and IOs. These parameters may correlate better with clinical disease and with certain exposures than simple spirometry (50, 51).

Although our exposure groups were numerically imbalanced, these participants all came from a registry of people who suffered an injury during service in Iraq or Afghanistan. This yielded a pool of study participants with similar characteristics. This helped to limit impacts of a healthy warrior effect, wherein military populations, because of requirements for military service or deployment, are ultimately healthier compared to the general population, or wherein those deployed are healthier than those who did not deploy. Additionally, veterans do not self-select to join the TEF Registry. They are automatically added to the TEF Registry based on responses to questions administered by a VA provider (52). This also helps to mitigate selection bias, as our recruitment pool included participants included in the registry based on standardized clinical screening responses. Notably, however, we are unable to incorporate other measures that may impact respiratory health, such as systemic or post-traumatic stress (because it was not assessed) or other injury-related respiratory insults (because this was only assessed in relation to blast exposure), into comparisons between the blast unexposed and exposed groups. For example, we do not know if the blast unexposed veterans may have also experienced thoracic trauma, because the format of the question asked if the veteran experienced thoracic trauma from a blast. We know from a prior questionnaire-based study among TEF registrants that thoracic trauma from a blast or explosion, for example, was approximately 12% 0.1 (1). We hypothesize it would be unlikely that the blast unexposed group would have a significantly higher rate of thoracic trauma. Nevertheless, we are unable to account for this.

The findings of the current study highlight the need for consensus regarding the best epidemiologic approach for quantifying blast exposure, including measures of blast pressure waves and experienced doses. This type of evaluation is notoriously challenging due to the lack of objective measure of blast (for example, use of pressure monitors) and confounding simultaneous exposures (6, 48, 53). Ideally, the tools used for collecting these data would differentiate between acute high- or low-level blast and total cumulative blast (54). Additionally, mechanistic research to understand associations between TBI and respiratory health effects is needed, potentially discriminating between blast-induced and impact-induced (55). Given the prevalence of TBI in post 9/11 military populations, TBI and blast should be assessed in studies evaluating respiratory health in addition to inhalational exposures, such as from particulate matter, combustion products, allergens, and metals (6, 11).

In summary, we observed significantly more dyspnea in post 9/11 veterans who self-reported blast exposure, although we did not observe lower lung function values compared to those without blast exposure. We also observed associations between lower lung volume measurements among blast-exposed post 9/11 veterans with diagnoses of TBI compared to those without TBI. These patterns persist regardless of other significant predictors of adverse respiratory outcomes, such as tobacco exposure and exposure to dust. Future studies should further investigate mechanistic hypotheses for respiratory disease associated with TBI and validating best metrics for estimating blast exposure.

## Data Availability

The datasets presented in this article are not readily available because data from the study are not freely available for other investigators based on the language that was communicated with participants during the informed consent process. Requests to access the datasets should be directed to shines@som.umaryland.edu.
